# Repeat stereotactic radiosurgery for progressive vestibular schwannomas after primary gamma knife radiosurgery

**DOI:** 10.1007/s11060-024-04761-9

**Published:** 2024-07-29

**Authors:** Suchet Taori, Othman Bin-Alamer, Anthony Tang, Ajay Niranjan, John C. Flickinger, Constantinos G. Hadjipanayis, L. Dade Lunsford

**Affiliations:** 1 School of Medicine, University of Pittsburgh Medical Center, Pennsylvania, PA USA; 2 Department of Neurological Surgery, University of Pittsburgh Medical Center, 200 Lothrop Street, Pennsylvania, PA 15213 USA; 3 Department of Radiation Oncology, University of Pittsburgh Medical Center, Pennsylvania, PA USA

**Keywords:** Stereotactic radiosurgery, Vestibular schwannoma, Tumor control, Hearing preservation, Facial neuropathy, Adverse radiation effect

## Abstract

**Purpose:**

Limited data provides guidance on the management of vestibular schwannomas (VSs) that have progressed despite primary Gamma Knife radiosurgery (GKRS). The present article reports our long-term experience after repeat GKRS for VS with sustained progression after solely primary GKRS management.

**Methods:**

A retrospective review of 1997 patients managed between 1987 and 2023 was conducted. Eighteen patients had sustained tumor progression after primary GKRS and underwent repeat GKRS. The median repeat GKRS margin dose was 11 Gy (IQR: 11–12), the median tumor volume was 2.0 cc (IQR: 1.3–6.3), and the median cochlear dose in patients with preserved hearing was 3.9 Gy (IQR: 3-4.1). The median time between initial and repeat GKRS was 65 months (IQR: 38–118).

**Results:**

The median follow-up was 70 months (IQR: 23–101). After repeat GKRS, two patients had further tumor progression at 4 and 21 months and required partial resection of their tumors. The 10-year actuarial tumor control rate after repeat GKRS was 88%. Facial nerve function was preserved in 13 patients who had House-Brackmann grade 1 or 2 function at the time of repeat GKRS. Two patients with serviceable hearing preservation (Gardner-Robertson grade 1 or 2) at repeat GKRS retained that function afterwards. In patients with tinnitus, vestibular dysfunction, and trigeminal neuropathy, symptoms remained stable or improved for 16/16 patients, 12/15 patients, and 10/12 patients, respectively. One patient developed facial twitching in the absence of tumor growth 21 months after repeat GKRS.

**Conclusions:**

Repeat GKRS effectively controlled tumor growth and preserved cranial nerve outcomes in most patients whose VS had sustained progression after initial primary radiosurgery.

## Introduction

Due to the high tumor control rates, few published series have examined the outcomes of vestibular schwannoma (VS) patients who have had repeat Gamma Knife radiosurgery (GKRS) because of sustained progression after primary GKRS. [1–3]. Albano et al. confirmed that regardless of tumor size ranging from Koos Grade 1 to Koos Grade 4, transient enlargement may be seen for 9.4% of patients who undergo primary GKRS [4]. The need for additional management, therefore, is based on the development of sustained tumor progression over 2–3 years after primary GKRS. Both microsurgical resection and repeat GKRS have been advocated as salvage approaches [5–11]. Much of the published repeat GKRS vestibular schwannoma literature includes patients with heterogeneous treatment histories, including those who failed surgical resection prior to initial GKRS [5–8]. In this focused single-institution experience, we report the outcomes after repeat GKRS for VS with sustained progression after solely primary initial GKRS.

## Methods

### Patient inclusion criteria and data parameters

Using our prospectively maintained data base, a 36-year retrospective review of 1997 VS patients who underwent primary or post-surgical resection salvage Gamma Knife GKRS was performed. Patients were excluded if they had surgical resection prior to GKRS, or neurofibromatosis type 2 (NF-2), the outcomes of which were analyzed separately [12]. We identified 18 patients who underwent repeat (salvage) GKRS after serial imaging studies confirmed continued tumor progression. Eight of these 18 patients elected for repeat GKRS after sustained and continuous VS growth, despite large tumor volumes, due to advanced age, symptom severity, and/or the presence of multiple comorbidities that rendered them poor surgical candidates. Patient characteristics, including patient sex, age, prior management, clinical symptoms, pertinent audiometric data, initial and repeat GKRS treatment parameters and clinical outcomes, were evaluated.

### GKRS technique

The GKRS procedure has been documented in previous publications [13, 14]. Briefly, a Leksell stereotactic headframe was applied to each patient with intravenous sedation and local anesthetic administration. In MRI eligible patients, high-resolution T1-weighted brain magnetic resonance imaging (MRI) sequences (1.5 mm slice) with contrast (gadolinium) were acquired for each patient following the placement of the stereotactic frame. Additional axial fast spin echo T2-weighted images were used to refine outlining of the tumor boundary and adjacent cranial nerves. The whole tumor volume of the progressed VS was targeted, unless the demarcation of the progressed portion was clearly delineated, allowing for a target volume focusing on the progressed portion only. In selected patients, computed tomography imaging was used as an alternative or additional imaging modality. Specific Gamma Knife Models U, B, C, 4 C, Perfexion, and ICON (Elekta, AB) were utilized depending on the 36-year interval. Dose plans for all identifiable tumors were created using various iterations of the GammaPlan software. Procedures were performed under the direction of a neurosurgeon, a radiation oncologist, and a medical physicist.

### Radiographic and clinical follow Up and study endpoints

Patients had clinical and radiological follow-up typically at 6-, 12-, 24-, 48- months, and then every 4 years. Tumor volumes and clinical findings were studied to determine responses that included regression (tumor volumetric reduction of > 20%), stability (tumor volume within ± 20% of the retreatment volume), or sustained tumor volumetric progression. Sustained tumor progression was defined as persistent and continuing growth of VS across at least two consecutive radiological evaluations following the initial GKRS, and that eventually exceeded more than 20% of the tumor volume at the time of initial GKRS with symptom worsening or development. Tumors exhibiting initial progression that subsequently stabilized were observed. Transient tumor enlargement, defined as gradually increasing tumor volume followed by volume stabilization or shrinkage, was not considered as true VS tumor progression. The primary endpoint of this study was tumor control after salvage GKRS. Secondary endpoints included new neurological symptoms or sign development and adverse radiation effects (ARE). ARE were defined as the development of symptomatic peri-tumoral reactive edema as defined by T2 MRI changes, or the development of new or worsened clinical symptoms in the absence of tumor progression. Actuarial tumor response was calculated starting from the repeat GKRS date. Neurological symptom comparison included neurological symptoms before and after the repeat GKRS. In patients with preserved hearing, the Gardner-Robertson scale was used to assess hearing [15]. To assess facial nerve function, the House-Brackmann Scale was used to evaluate facial nerve function [16]. Other clinical symptoms assessed included tinnitus, balance, or equilibrium disorders (vestibulopathy), and trigeminal nerve dysfunction.

### Statistical analysis

Excel version 2022 (Microsoft, Washington) was utilized to perform basic calculations. Kaplan-Meier curves were created on Prism version 9 (GraphPad, California) and were used to plot tumor control. Univariable Cox proportional hazard analyses were performed using the *survival* package in R version 4.2.1 (RStudio, Massachusetts). Continuous variables were summarized as medians and IQR (25%ile-75%ile), while categorical variables were summarized as counts and percentages. A *p* < 0.05 was considered statistically significant for all analyses.

## Results

### Patient demographics and GKRS management characteristics

Eighteen (10 male) VS patients were included in this study **(**Table 1**)**. Prior to the initial GKRS, no patient had undergone partial or complete resection or radiation therapy. At initial GKRS, the median age was 54 years (IQR: 44–63). Two patients had Koos grade 1 tumors, 8 had Koos grade 2 tumors, 2 patients had Koos grade 3, and 4 had Koos grade 4. The Koos grade was unavailable for 2 patients at the initial GKRS as these two patients were part of the early experience and imaging were not available in the medical records. The median margin dose for the initial GKRS was 12.5 Gy (IQR: 12–13), and the maximum dose was 25 Gy (IQR: 24–26). The median tumor volume at the initial GKRS was 1.1 cc (IQR: 0.5–2.6), and the median isodose was 50% (IQR: 50–50). In this series, sustained tumor progression was documented at a median of 64 months (IQR: 33–118) after initial radiosurgery **(**Table 1**)**. Repeat GKRS was performed within three months of the confirmation of sustained tumor progression. Four patients had repeat GKRS within 36 months of the initial GKRS after documented progression on at least 2 follow up visits. Three of these 4 patients had documented radiographic progression with worsening of symptoms and 1 patient had radiographic progression with new symptom development. Due to advanced age and multiple comorbidities, these four patients elected for repeat GKRS. The remaining 14 patients were treated 36 months after the initial GKRS. At repeat GKRS, 14 patients were treated for tumor growth causing worsening neurologic symptoms and 4 patients were treated for tumor enlargement causing new neurologic deficits. Between initial and repeat GKRS, one patient also underwent subtotal resection 2 months before repeat GKRS. At the time of repeat GKRS, the median patient age was 60 years (IQR: 51–70). One patient had a Koos grade 1 tumor, 3 had Koos grade 2 tumors, 6 patients had Koos grade 3, and 8 had Koos grade 4. The median margin and maximum doses at the repeat GKRS were 11 Gy (IQR: 11–12) and 23 Gy (IQR: 22–24), respectively, at a 50% (IQR: 50–50) median isodose. The median tumor volume was 2.0 cc (IQR: 1.3–6.3), and the median average cochlear dose in patients with preserved hearing at the time of repeat GKRS was 3.9 Gy (IQR: 3-4.1).

**Table 1:** Patient characteristics, SRS management parameters, and radiographic outcomes

Patient Characteristics	Value	
Total number of pts.	18	
Male	10 (56%)	
Location of VS (no. of pts.)		
Right sided	8 (44%)	
Left sided	10 (56%)	
**Management Characteristics**	**Initial SRS Value**	**Repeat SRS Value**
Surgery prior to initial SRS		
None	18 (100%)	-
Age at SRS (yrs.)	54 [44–63]	60 [51–70]
Koos Grade (no. of patients)		
1	2 (11%)	1 (6%)
2	8 (44%)	3 (17%)
3	2 (11%)	6 (33%)
4	4 (22%)	8 (44%)
Unavailable	2 (11%)	0 (0%)
Margin dose (Gy)	12.5 [12–13]	11 [11–12]
Maximum dose (Gy)	25 [24–26]	23 [22–24]
Mean cochlear dose (Gy)	4.7 [3.4–5.7]	3.9 [3–4.1]
Median isodose (%)	50 [50–50]	50 [50–50]
Tumor volume (cc)	1.1 [0.5–2.6]	2.0 [1.3–6.3]
Surgery between SRS (no. of pts.)		
Subtotal resection	-	1 (6%)
Time from initial SRS to progression (mo.)	64 [33–118]	-
Time from initial SRS to repeat SRS (mo.)	65 [38–118]	-
Time from repeat SRS to last follow-up (mo.)	-	37 [20–79]
Volumetric tumor response after repeat SRS (no. of pts.)		
Regression	-	7 (39%)
Stable	-	9 (50%)
Progression	-	2 (11%)
Time from repeat SRS to progression (mo.)	-	13 [4–21]
Management for progression (no. of pts.)	-	
Surgical resection	-	2 (12%)
Hydrocephalus after SRS	1 (6%)	0 (0%)
Adverse Radiation Effects after SRS	0 (0%)	1 (6%)
No. of patients deceased at follow-up	-	2 (11%)

SRS = stereotactic radiosurgery. Pts. = patients. No. = number. Mo. = months. Yrs. = years. Table values are represented as either number (%), or median [inter-quartile range]

### Tumor control after repeat GKRS

**Fig. 1:**
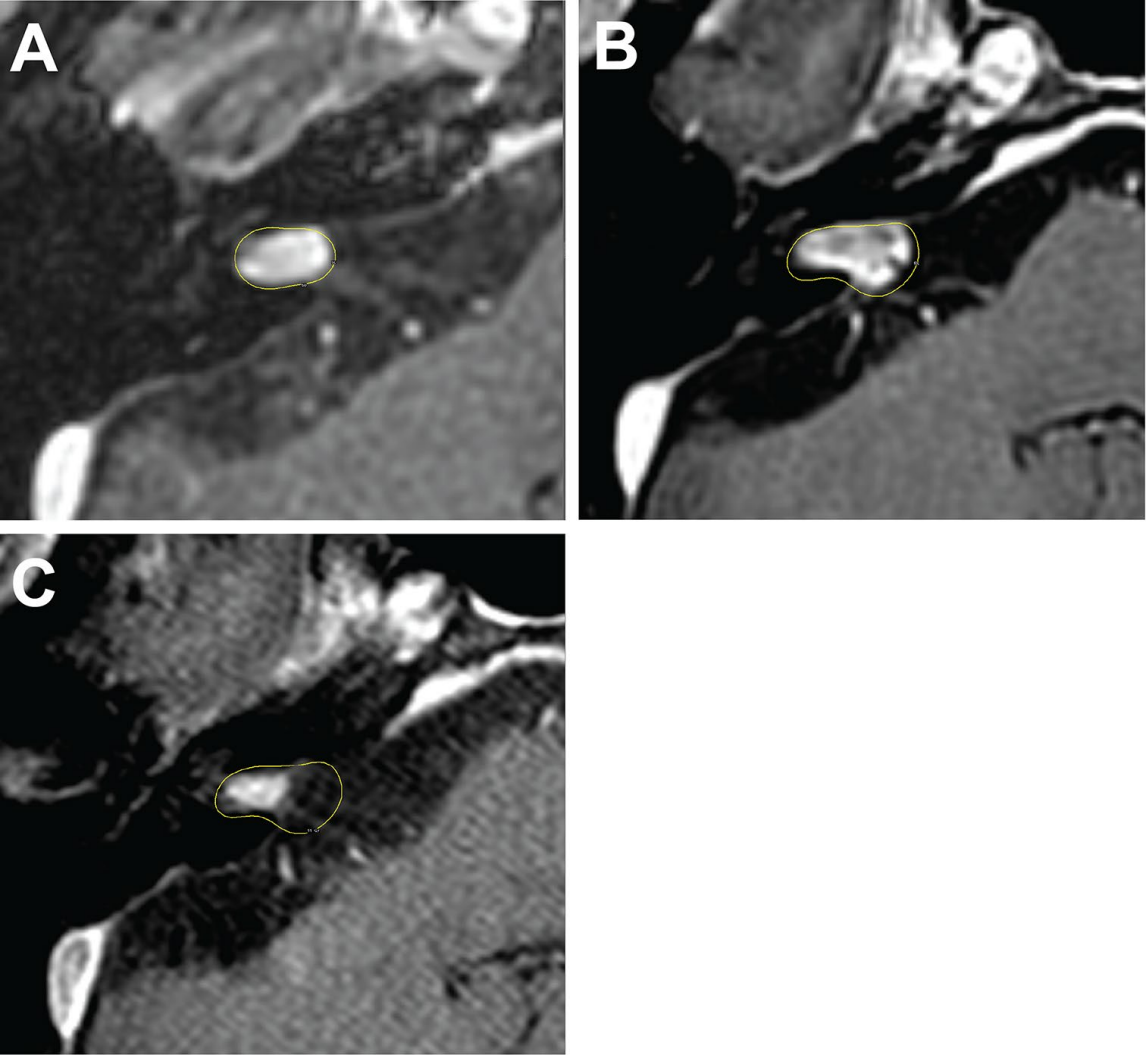
A 67-year-old male presenting with tinnitus and serviceable hearing underwent primary GKRS for a right sided vestibular schwannoma, sized 0.19 cc, as depicted in the axial and sagittal MRI sequence (Panel A). The tumor was initially targeted with a margin dose of 12 Gy at 50% isodose. The patient was serially monitored until developed persist tumor progression (0.42 cc) at 53 months after initial GKRS (Panel B). At this time, the patient’s tinnitus and serviceable hearing remained stable. The tumor in Panel B was managed with a tumor margin dose of 11 Gy at 55% isodose and a mean cochlear dose of 4 Gy. At last follow-up (Panel C), 47 months after the repeat GKRS, the patient’s vestibular schwannoma size had regressed (0.15 cc), and all clinical symptoms, including serviceable hearing, remained stable

**Fig. 2:**
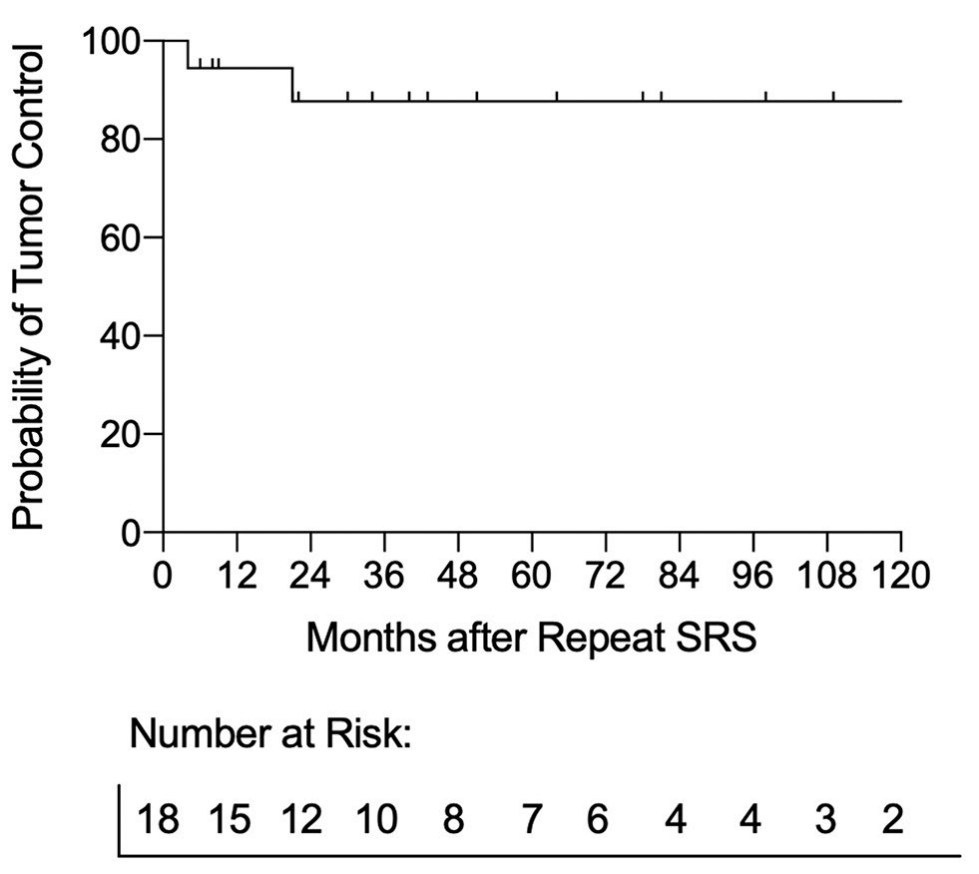
**Tumor control.** Tumor control response of the repeat VS. The 1-, 5- and 10- year tumor control probabilities are 94%, 88%, and 88%, respectively

The median radiological and clinical follow-up interval after repeat GKRS were 37 months (IQR: 20–79) and 70 months (IQR: 23–101), respectively. At their last follow-up, seven patients had tumor regression, 9 had stable tumor volumes, and 2 patients had continued tumor progression 4 and 21 months after repeat GKRS **(**Table 1 and Fig. 1**)**. Both patients with continued tumor progression underwent delayed partial surgical resection for their true tumor progression and associated brainstem compression that affected cranial nerve function. The histopathology results for these 2 patients indicated a World Health Organization (WHO) grade 1 vestibular schwannoma with a Ki-67 < 5%. The 1-, 5-, and 10-year actuarial tumor control rates after repeat GKRS were 94%, 88%, and 88%, respectively **(**Fig. 2**)**. No clinical factor showed a significant association with tumor progression (Table 2**)**. At last follow-up after repeat GKRS, 2 patients had died from causes unrelated to their VS **(**Table 1**)**.

**Table 2:** Univariable and multivariable cox proportional hazards for tumor control after repeat SRS

	Univariable		
	HR	95% CI	*P*
*Tumor control*			
Age	1.12	0.94–1.34	0.2
Female	1.29	0.08–20.6	0.9
Time between SRS	0.98	0.94–1.03	0.4
Margin dose	0.20	0.03–1.26	0.086
Max dose	0.68	0.29–1.61	0.4
VS volume	1.38	0.82–2.33	0.2

HR = Hazard ratio. 95% CI = 95% confidence interval. A *P* < 0.05 was considered significant. All prognostic factors are continuous or binary variables

### Clinical outcomes after repeat GKRS

One (6%) patient developed ARE. This patient developed worsened facial, trigeminal, and vestibular neuropathies 21 months after repeat GKRS in the absence of tumor progression and peritumoral edema on imaging. This patient was initially treated with oral vitamin E and Pentoxifylline without benefit. Fifteen cycles of bevacizumab were administered with slight improvement in the patient’s symptoms. The patient still has facial spasms, but less frequently, and is currently being managed with observation. No patient developed hydrocephalus or malignant tumor transformation after repeat GKRS **(**Table 1**)**.

At the time of repeat GKRS, 2 patients had serviceable hearing (GR grade 1 or 2) **(**Table 3**)**. Both patients maintained serviceable hearing status after repeat GKRS. At the time of repeat GKRS, 6 patients exhibited various degrees of facial weakness (HB Grade 2–6). After repeat GKRS, the HB grade improved in 2 patients, remained stable in 1 patient, and worsened in 3 patients. In two patients, worsening facial neuropathy was related to continued tumor progression and both underwent surgical resection. Both patients exhibited HB grade 1 at initial and repeat GKRS and HB grade 2 before surgical resection. One patient had worsening facial neuropathy in the absence of tumor progression or peritumoral edema (as described above). This patient had HB grade 1 at initial and repeat GKRS, and HB grade 4 at last follow-up. At last follow-up, 13 out of 14 patients with initial HB grades of 1 or 2 maintained HB grades of 1 or 2 at their last follow-up after repeat GKRS. (Table 3).

**Table 3:** Hearing and facial nerve function over time

Characteristics	At Initial SRS	At Repeat SRS	At Last Follow-up
Gardner-Robertson Grade (no. of patients)			
1	6 (33%)	2 (11%)	1 (6%)
2	5 (28%)	0 (0%)	1 (6%)
3	2 (11%)	4 (22%)	3 (17%)
4	0 (0%)	4 (22%)	2 (11%)
5	5 (28%)	8 (44%)	11 (61%)
House-Brackmann Grade (no. of patients)			
1	15 (83%)	12 (67%)	9 (50%)
2	2 (11%)	2 (11%)	5 (28%)
3	0 (0%)	1 (6%)	2 (11%)
4	0 (0%)	3 (17%)	2 (11%)
5	0 (0%)	0 (0%)	0 (0%)
6	0 (0%)	0 (0%)	0 (0%)
Unavailable	1 (6%)	0 (0%)	0 (0%)

SRS = stereotactic radiosurgery. No. = number. Table values are represented as number (%)

**Fig. 3:**
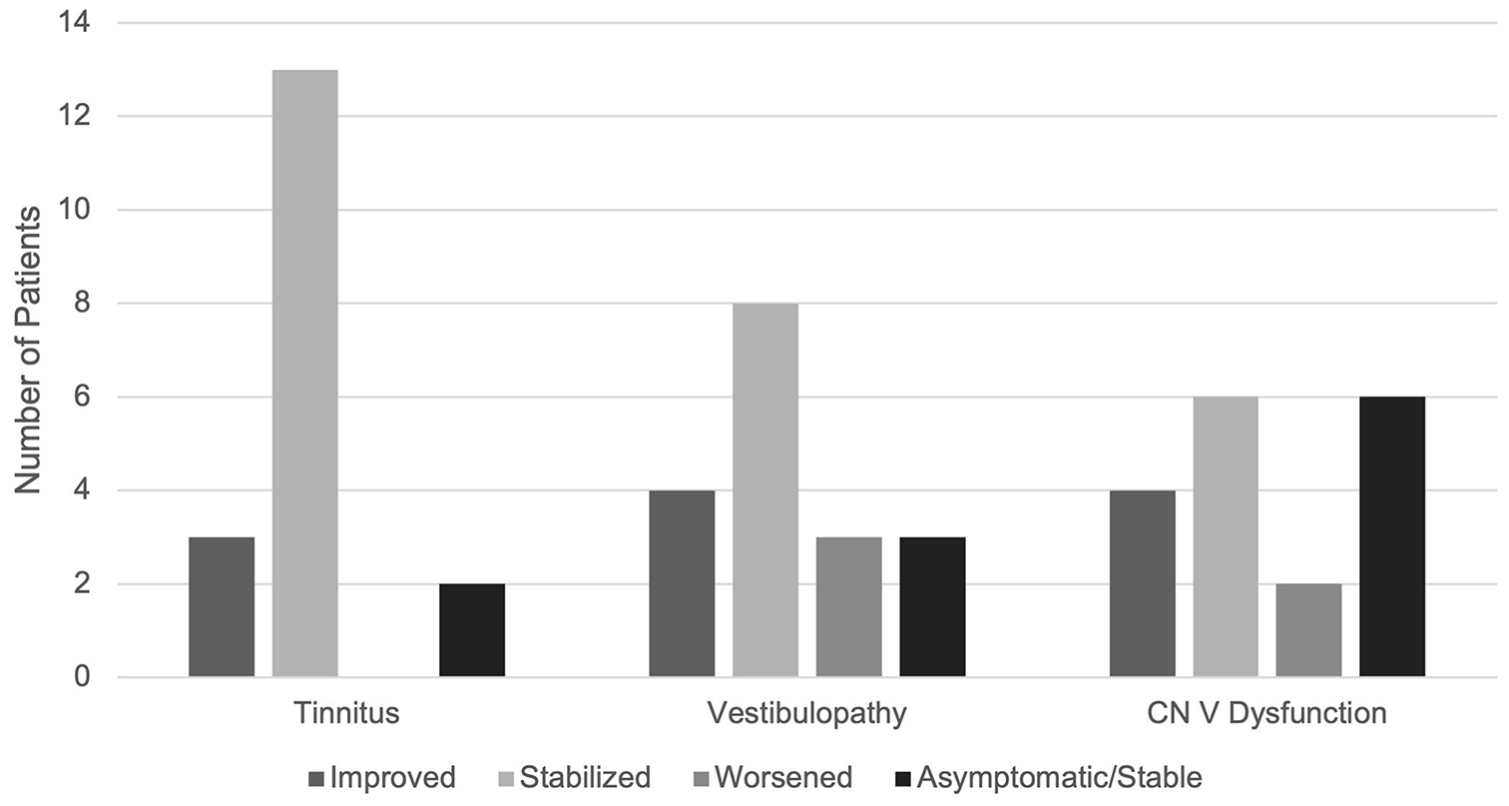
**Clinical symptom changes after repeat GKRS.** Changes in tinnitus, vestibulopathy, and CN V dysfunction, comparing symptoms at the repeat GKRS with symptoms after the repeat GKRS. Asymptomatic/Stable refers to patients who did not have associated symptoms at the time of repeat GKRS and continued to remain symptom-free at last follow-up

Twelve patients had ipsilateral trigeminal neuropathy at the time of repeat GKRS **(**Fig. 3**)**. Trigeminal symptoms improved in 4 patients, worsened in 2 (1 from tumor progression, 1 from radiation effects), and stabilized in 6. The patient with tumor progression-related trigeminal neuropathy underwent tumor resection and had symptom stability at last follow-up. Tinnitus was reported in 16 patients at the time of repeat GKRS. Tinnitus improved in 3 patients and remained stable in 13 patients. Vestibular dysfunction was present in 15 patients at the time of repeat GKRS. After repeat GKRS, vestibular function improved in 4, worsened in 3 (2 from tumor progression, 1 from radiation effects [same patient described above]), and stabilized in 8. The two patients with tumor progression-related vestibular symptom exacerbation underwent surgical resection, achieving symptom stability at last follow-up. No other patients developed new trigeminal nerve dysfunction, tinnitus, or vestibulopathy after the repeat GKRS (Fig. 3).

## Discussion

Due to the rarity of tumor progression after GKRS management of VS, relatively few studies have examined patient outcomes after repeat GKRS for sustained tumor progression [5, 8–10, 22]. None have solely reported outcomes on patients who had primary GKRS. The present study builds on our 2010 study that evaluated the role of repeat GKRS for persistently enlarging VS [6]. Compared to the 2010 study, the current study limits the objective to outcomes after repeat GKRS for primary GKRS, expands the sample size from 6 to 18, reports an increased clinical follow-up duration from 29 to 70 months, and examines prognostic factors associated with tumor control following the repeat GKRS. After repeat GKRS We found that the 10-year actuarial tumor control rate was 88% and the combined risk of new cranial nerve dysfunction and ARE was 6%.

### Repeat GKRS for progressive VS after initial GKRS

Although primary GKRS for VS is associated with excellent tumor control, failure and progression have been reported in 2–5% of cases [3, 23, 24]. The time from initial GKRS to persistent VS progression has been reported to range between 12 and 185 months [5–8]. In the present series, the median time from the initial GKRS to the recognition of sustained progression was 64 (IQR: 33–118) months. This extended time interval after the initial GKRS underscores the importance of long-term follow-up after GKRS. Repeat GKRS provides high tumor control rates while minimizing major complications [5, 7, 8, 25]. During a 37-month median follow-up after the repeat GKRS, we found a 10-year tumor control rate of 88%. In cases of asymptomatic tumor progression that followed by volume stabilization or shrinkage, we advocate for observation. In patients with sustained and continuing tumor progression over at least two consecutive radiological assessments with worsening or new neurologic symptom development, we advise a case-by-case approach in which patients with small/medium size tumors, undergo repeat GKRS. However, emergent cases with severe symptoms, large tumors, and rapid, sustained and continuing progression along with good surgical candidacy, should undergo microsurgical resection.

Iorio-Morin et al. reported 76 heterogeneously treated patients who underwent repeat GKRS for tumor progression [5]. With a median margin dose of 12 Gy, they also reported higher 5- and 10-year tumor control rates of 92.2% after the repeat GKRS. At a median follow-up time of 75 months after the repeat GKRS, Fu et al. reported 100% tumor control [8]. They utilized a median margin dose of 11.8 Gy. Lonneville et al. managed 27 patients [7]. At a median follow-up time of 46 months, they reported an 85% tumor control rate after repeat GKRS. At a median follow-up of 43 months after repeat GKRS, Liscak et al. reported a tumor control rate of 92.3% [25].

Prior reports have found that lower mean cochlear doses are significantly associated with preserved hearing function [23-25]. While there remains no well-defined consensus on the optimal cutoff dose for the mean cochlear dose, many studies and organizational guidelines recommend a mean dose to the cochlea of < 4 Gy to preserve hearing [17, 19]. In the present study, the median margin dose was 11 Gy, and the median mean cochlear dose was 3.9 Gy. In the 2 patients who maintained serviceable hearing after the repeat GKRS, the median mean cochlear doses were 3.9 and 4 Gy at the repeat GKRS. Two patients had serviceable hearing at the repeat GKRS; after repeat GKRS, both patients retained useful hearing. Iorio-Morin et al. reported useful hearing in 30%, 8%, and 5% of patients at initial GKRS, repeat GKRS, and last follow-up [5]. Liscak et al. reported that 1 patient with useful hearing at repeat GKRS lost useful hearing after the repeat GKRS [25]. Lonneville et al. reported that 5 patients with useful hearing at the repeat GKRS lost useful hearing after the repeat GKRS [7]. However, they utilized a median margin dose of 12 Gy. Our data, in addition to other published studies, showcase the importance of precise dose planning and illustrate the benefit of considering doses lower than what was delivered in initial GKRS procedures.

Preserving facial nerve function is of high importance, particularly in patients with no existing facial palsy, and is challenging for all current management approaches for progressive VS. In the present study, 14 patients had a HB grade 1 or 2 at the repeat GKRS. After the repeat GKRS, 13 patients maintained a HB grade 1 or 2. Our results were in line with other published data demonstrating high preservation rate (95–100%) of useful (HB grade 1 or 2) facial nerve function after the repeat GKRS. [6, 7, 22].

### Adverse radiation effects after repeat GKRS

In the present study, one patient developed ARE (worsened facial twitching and weakness, decreased sensation, and vestibular dysfunction) in the absence of tumor progression after repeat GKRS. This patient had no prior surgical management, no tumor progression after the repeat GKRS, and no peritumoral edema on imaging. No cases of hydrocephalus after the repeat GKRS or malignant transformations were reported in this series. Malignant transformations of VSs are extremely rare in the literature, with reports indicating VS rates of transformation of 0.02% [17, 21]. However, both patients in this series who progressed after repeat GKRS were confirmed to have WHO Grade 1 VS on pathology reports with Ki-67% < 5%. Iorio-Morin et al. [5] reported no patients who developed tumor radiation necrosis or radiation-induced edema but 4 patients who developed hydrocephalus and required VP shunting. Fu et al. and Dewan & Noren reported on 1 (3.6%), and 2 (18%) patients, respectively, who developed symptomatic radiation-induced edema [8, 18]. Although rare, ARE risks for patients undergoing a repeat GKRS do exist and must be weighed carefully in the context of patients’ overall clinical condition.

### Surgical resection for progressive VS after GKRS

Various management strategies have been adopted to manage VS that have sustained tumor growth despite initial GKRS [5, 8–10, 22]. Salvage surgical resection procedures include both retrosigmoid and translabyrinthine approaches. After either total or partial resection, subsequent tumor control rates are high but are associated with risks that include stroke, CSF leak, and infection-related complications [2]. The translabyrinthine approach, utilized in nearly 50% of cases, results in complete loss of hearing [19]. Friedman et al. reported 73 patients who underwent delayed microsurgery for VS progression after GKRS [9]. Ten (13.7%) patients had complete facial nerve palsies and only 36 (58%) patients maintained a HB grade 1 or 2 after surgical resection. Nonaka et al. reported difficulty in dissecting 27 (69.2%) VS tumors after prior GKRS and reported that almost 20% of patients had new facial nerve palsies after resection [10]. The timing of resection relative to the timing and type of the GKRS procedure in these patients is not clear.

### Limitations

This study is inherently limited due to its retrospective design and represents the experience of a single institution. Patients were managed across multiple decades during which GKRS models and dose planning techniques evolved. For VSs that progressed after the initial GKRS, but did not undergo any further surgical resection, the histopathology of the tumor could not be definitively verified. Future multi-center, higher-powered, and longer-termed prospective studies are warranted to optimize an GKRS management paradigm and determine associated significant prognostic factors for VS patients who demonstrate progressive tumor on imaging after GKRS management. Additionally, further studies may evaluate facial and trigeminal nerve dose tolerance to identify correlations between nerve dose tolerance and nerve function following GKRS.

## Conclusions

The present study emphasizes the effectiveness and safety of repeat GKRS for managing VS that have progressed following initial GKRS treatment. VS tumors that have progressed after initial GKRS may benefit from high rates of tumor control and cranial nerve preservation with repeat GKRS management. Our findings underscore the importance of urgent repeat GKRS as a primary intervention for progressed VS to prevent significant tumor enlargement and deterioration of the patient’s condition, situations where surgical resection may become necessary.

## Data Availability

No datasets were generated or analysed during the current study.

## Abbreviations

GKRS: stereotactic radiosurgery
VS: vestibular schwannoma
GR: Gardner-Robertson
HB: House-Brackmann

## References

[1] Balossier A, Regis J, Reyns N, Roche PH, Daniel RT, George M, Faouzi M, Levivier M, Tuleasca C (2021) Repeat stereotactic radiosurgery for progressive vestibular schwannomas after previous radiosurgery: a systematic review and meta-analysis. Neurosurg Rev 44:3177–3188. 10.1007/s10143-021-01528-y 33847846 10.1007/s10143-021-01528-y PMC8592961

[2] Whitmeyer M, Brahimaj BC, Beer-Furlan A, Alvi S, Epsten MJ, Crawford F, Byrne RW, Wiet RM (2020) Resection of vestibular schwannomas after stereotactic radiosurgery: a systematic review. J Neurosurg 135:881–889. 10.3171/2020.7.JNS2044 34331121 10.3171/2020.7.JNS2044

[3] Kondziolka D, Lunsford LD, McLaughlin MR, Flickinger JC (1998) Long-term outcomes after radiosurgery for acoustic neuromas. N Engl J Med 339:1426–1433. 10.1056/NEJM199811123392003 9811917 10.1056/NEJM199811123392003

[4] Albano L, Deng H, Wei Z, Vodovotz L, Niranjan A, Lunsford LD (2023) The longitudinal volumetric response of vestibular schwannomas after Gamma Knife radiosurgery. J Neurosurg 138:1273–1280. 10.3171/2022.7.JNS22812 36087328 10.3171/2022.7.JNS22812

[5] Iorio-Morin C, Liscak R, Vladyka V, Kano H, Jacobs RC, Lunsford LD, Cohen-Inbar O, Sheehan J, Emad R, Karim KA, El-Shehaby A, Reda WA, Lee CC, Pai FY, Wolf A, Kondziolka D, Grills I, Lee KC, Mathieu D (2019) Repeat stereotactic radiosurgery for Progressive or recurrent vestibular Schwannomas. Neurosurgery 85:535–542. 10.1093/neuros/nyy416 30189018 10.1093/neuros/nyy416

[6] Kano H, Kondziolka D, Niranjan A, Flannery TJ, Flickinger JC, Lunsford LD (2010) Repeat stereotactic radiosurgery for acoustic neuromas. Int J Radiat Oncol Biol Phys 76:520–527. 10.1016/j.ijrobp.2009.01.076 19783373 10.1016/j.ijrobp.2009.01.076

[7] Lonneville S, Delbrouck C, Renier C, Devriendt D, Massager N (2015) Repeat Gamma Knife surgery for vestibular schwannomas. Surg Neurol Int 6:153. 10.4103/2152-7806.166173 26500799 10.4103/2152-7806.166173 PMC4596053

[8] Fu VX, Verheul JB, Beute GN, Leenstra S, Kunst HPM, Mulder JJS, Hanssens PEJ (2018) Retreatment of vestibular schwannoma with Gamma Knife radiosurgery: clinical outcome, tumor control, and review of literature. J Neurosurg 129:137–145. 10.3171/2017.3.JNS162033 28984523 10.3171/2017.3.JNS162033

[9] Friedman RA, Berliner KI, Bassim M, Ursick J, Slattery WH 3rd, Schwartz MS, Brackmann DE (2011) A paradigm shift in salvage surgery for radiated vestibular schwannoma. Otol Neurotol 32:1322–1328. 10.1097/MAO.0b013e31822e5b76 21897324 10.1097/MAO.0b013e31822e5b76

[10] Nonaka Y, Fukushima T, Watanabe K, Friedman AH, Cunningham CD 3rd, Zomorodi AR (2016) Surgical management of vestibular schwannomas after failed radiation treatment. Neurosurg Rev 39:303–312 discussion 312. 10.1007/s10143-015-0690-7 26782633 10.1007/s10143-015-0690-7

[11] Wise SC, Carlson ML, Tveiten OV, Driscoll CL, Myrseth E, Lund-Johansen M, Link MJ (2016) Surgical salvage of recurrent vestibular schwannoma following prior stereotactic radiosurgery. Laryngoscope 126:2580–2586. 10.1002/lary.25943 27107262 10.1002/lary.25943

[12] Mathieu D, Kondziolka D, Flickinger JC, Niranjan A, Williamson R, Martin JJ, Lunsford LD (2007) Stereotactic radiosurgery for vestibular schwannomas in patients with neurofibromatosis type 2: an analysis of tumor control, complications, and hearing preservation rates. Neurosurgery 60: 460–468; discussion 468–470 10.1227/01.NEU.0000255340.26027.53 10.1227/01.NEU.0000255340.26027.53 17327790

[13] Bin-Alamer O, Faramand A, Alarifi NA, Wei Z, Mallela AN, Lu VM, Nabeel AM, Reda WA, Tawadros SR, Abdelkarim K, El-Shehaby AMN, Emad RM, Peker S, Samanci Y, Lee CC, Yang HC, Delabar V, Mathieu D, Tripathi M, Kearns KN, Bunevicius A, Sheehan JP, Chytka T, Liscak R, Moreno NM, Alvarez RM, Grills IS, Parzen JS, Cifarelli CP, Rehman AA, Speckter H, Niranjan A, Lunsford LD, Abou-Al-Shaar H (2023) Stereotactic radiosurgery for Vestibular Schwannoma in neurofibromatosis type 2: an International Multicenter Case Series of Response and Malignant Transformation Risk. Neurosurgery 92:934–944. 10.1227/neu.0000000000002436 36861994 10.1227/neu.0000000000002436 PMC10079356

[14] Pikis S, Mantziaris G, Kormath Anand R, Nabeel AM, Sheehan D, Sheehan K, Reda WA, Tawadros SR, Abdelkarim K, El-Shehaby AMN, Emad Eldin R, Peker S, Samanci Y, Kaisman-Elbaz T, Speckter H, Hernandez W, Isidor J, Tripathi M, Madan R, Zacharia BE, Daggubati LC, Martinez Moreno N, Martinez Alvarez R, Langlois AM, Mathieu D, Deibert CP, Sudhakar VR, Cifarelli CP, Arteaga Icaza D, Cifarelli DT, Wei Z, Niranjan A, Barnett GH, Lunsford LD, Bowden GN, Sheehan JP (2023) Stereotactic radiosurgery for Koos grade IV vestibular schwannoma: a multi-institutional study. J Neurosurg 138:405–412. 10.3171/2022.4.JNS22203 36303474 10.3171/2022.4.JNS22203

[15] Gardner G, Robertson JH (1988) Hearing preservation in unilateral acoustic neuroma surgery. Ann Otol Rhinol Laryngol 97:55–66. 10.1177/000348948809700110 3277525 10.1177/000348948809700110

[16] House JW, Brackmann DE (1985) Facial nerve grading system. Otolaryngol Head Neck Surg 93:146–147. 10.1177/019459988509300202 3921901 10.1177/019459988509300202

[17] Behling F, Bersali I, Santacroce A, Hempel J, Kandilaris K, Schittenhelm J, Tatagiba M (2022) Transition of a vestibular schwannoma to a malignant peripheral nerve sheath tumor with loss of H3K27 trimethylation after radiosurgery-a case report and review of the literature. Neurosurg Rev 45:915–922. 10.1007/s10143-021-01620-3 34392463 10.1007/s10143-021-01620-3 PMC8827336

[18] Dewan S, Noren G (2008) Retreatment of vestibular schwannomas with Gamma Knife surgery. J Neurosurg 109 Suppl:144–148. 10.3171/JNS/2008/109/12/S22 19123901 10.3171/JNS/2008/109/12/S22

[19] Roche PH, Pellet W, Moriyama T, Thomassin JM (2008) Translabyrinthine approach for vestibular schwannomas: operative technique. Prog Neurol Surg 21:73–78. 10.1159/000156708 18810201 10.1159/000156708

[20] Gerganov VM, Giordano M, Samii A, Samii M (2012) Surgical treatment of patients with vestibular schwannomas after failed previous radiosurgery. J Neurosurg 116:713–720. 10.3171/2011.12.JNS111682 22264180 10.3171/2011.12.JNS111682

[21] Seferis C, Torrens M, Paraskevopoulou C, Psichidis G (2014) Malignant transformation in vestibular schwannoma: report of a single case, literature search, and debate. J Neurosurg 121 Suppl:160–166. 10.3171/2014.7.GKS141311 25434949 10.3171/2014.7.GKS141311

[22] Yomo S, Arkha Y, Delsanti C, Roche PH, Thomassin JM, Regis J (2009) Repeat gamma knife surgery for regrowth of vestibular schwannomas. Neurosurgery 64:48–54 discussion 54– 45. 10.1227/01.NEU.0000327692.74477.D5 19050660 10.1227/01.NEU.0000327692.74477.D5

[23] Johnson S, Kano H, Faramand A, Pease M, Nakamura A, Hassib M, Spencer D, Sisterson N, Faraji AH, Arai Y, Monaco E, Niranjan A, Flickinger JC, Lunsford LD (2019) Long term results of primary radiosurgery for vestibular schwannomas. J Neurooncol 145:247–255. 10.1007/s11060-019-03290-0 31535315 10.1007/s11060-019-03290-0

[24] Soltys SG, Milano MT, Xue J, Tome WA, Yorke E, Sheehan J, Ding GX, Kirkpatrick JP, Ma L, Sahgal A, Solberg T, Adler J, Grimm J, El Naqa I (2021) Stereotactic radiosurgery for vestibular Schwannomas: Tumor Control Probability analyses and recommended reporting standards. Int J Radiat Oncol Biol Phys 110:100–111. 10.1016/j.ijrobp.2020.11.019 33375955 10.1016/j.ijrobp.2020.11.019 PMC9477217

[25] Liscak R, Vladyka V, Urgosik D, Simonova G, Vymazal J (2009) Repeated treatment of vestibular schwannomas after gamma knife radiosurgery. Acta Neurochir (Wien) 151:317–324 discussion 324. 10.1007/s00701-009-0254-0 19277457 10.1007/s00701-009-0254-0

[26] Ruess D, Pohlmann L, Grau S, Hamisch C, Hoevels M, Treuer H, Baues C, Kocher M, Ruge M (2020) Outcome and toxicity analysis of single dose stereotactic radiosurgery in vestibular schwannoma based on the Koos grading system. Sci Rep 10:9309. 10.1038/s41598-020-66213-4 32518238 10.1038/s41598-020-66213-4 PMC7283483

